# The Diagnostic Utility and Clinical Impact of After-Hours CT Scans of the Abdomen and Pelvis Investigating Abdominal Pain

**DOI:** 10.1155/2017/4028352

**Published:** 2017-12-14

**Authors:** Monil Karia, Matthew Seager, Akkib Rafique, Hemant Sheth

**Affiliations:** ^1^Department of Surgery and Cancer, Imperial College London, Kensington, London SW7 2AZ, UK; ^2^Department of Surgery, Ealing Hospital, Uxbridge Road, Southall UB1 3HW, UK

## Abstract

**Introduction:**

The aim of this study was to evaluate the diagnostic utility and impact on clinical management of after-hours CT scans investigating abdominal pain in surgical patients.

**Methods:**

After-hours CT A/P reports investigating the acute surgical abdomen were compared with clinical outcomes and histopathological findings to assess sensitivity and specificity of CT reporting. Comparisons between CT reports and clinical notes were made. CT scans were categorised as having direct effects on clinical management, ruling out a serious pathology, ruling out a nonserious pathology, or having no effect. Discrepancies between information in case-notes and information provided to radiologists were also analysed.

**Results:**

79 clinical notes were located. After-hours CT demonstrated 91% sensitivity and 82% reporting specificity using clinical outcomes as the standard. In the 26 patients with histopathological findings, CT reports demonstrated 91% sensitivity. In 79.7% of cases, CT scanning had an impact on management. In 35.4% of cases, an indication for scanning was not documented with variation in clinical information in 8.9% of cases.

**Discussion:**

This study demonstrates after-hours CT A/P reports result in significant impacts on clinical management of surgical patients with acute abdominal pain. Improvements in providing information when requesting scans are however needed to facilitate accurate reporting.

## 1. Introduction

Computed tomography of the abdomen and pelvis (CT A/P) is being increasingly used to investigate acute abdominal pain [1, 2]. This is in keeping with data from systematic reviews that have demonstrated excellent diagnostic accuracy of CT A/P for common acute surgical pathologies such as diverticulitis [3] and appendicitis [4, 5]. More specifically, in the setting of suspected acute appendicitis, CT has been shown to increase diagnostic certainty [6, 7] and impact on management [6, 8] and can reduce the rate of negative appendectomies [7–10]. Benefits have also been demonstrated for generalised abdominal pain, by making accurate diagnoses [11–15], impacting on management [12, 14–16], and reducing rates of admission [15].

There are few studies assessing the impact of CT A/P on mortality in the acute surgical abdomen. Ng et al. reported a reduction in mortality with the early use of CT versus standard management [17] and an England-wide study of high-risk surgical patients over 9 years found that greater institutional rates of CT use were an independent predictor of reduced mortality [18]. Whilst the potential benefit of CT use must be balanced against defined risks such as radiation exposure and contrast-induced nephropathy, CT A/P is a reliable tool in the work-up of the acute surgical abdomen.

To our knowledge, no studies have looked at the diagnostic impact and clinical utility of CT A/P to investigate acute abdominal pain in the after-hours setting. In the context of after-hours CT A/P for acute abdominal pain, the aim of this service evaluation project was thus threefold:

1. To evaluate the diagnostic utility of after-hours CT A/P by comparing image findings against clinical outcomes and, where possible, operative findings or histopathology
2. To assess the clinical impact of after-hours CT A/P reports on the patient's management
3. To evaluate the discrepancy in the clinical information, indication, and question provided to the radiologist compared to the documented clinical presentation.

## 2. Methods

This retrospective study was undertaken at a District General Hospital (DGH) in West London, with over 350 inpatient beds. After-hours radiology services included weekdays 17:00–09:00, weekends, and public holidays. Radiography and radiology services were similar within and after-hours, both being a consultant led service, with referrals made by senior house officer or registrar level clinicians within and after-hours. A book detailing all CT A/P scans performed after-hours kept in the Radiology Department was retrospectively hand-searched for scans referred by surgical teams to investigate acute abdominal pain, for the period of January–September 2013.

An electronic database of 96 consecutive surgical patients during this time period identified was generated and the Picture Archiving and Communication System (PACS) was used to locate the relevant scan reports as reported by consultant radiologists. Any scans which could not be completed or were of insufficient quality for interpretation were excluded. The scan reports were analysed before any other data was examined to summarise the reports into one of 6 categories: normal, acute inflammatory pathology, obstruction, perforation, miscellaneous, and inconclusive. Scans with unchanged findings reported on previous scans were considered normal.

The clinical information and question on the scan request form scanned into PACS were noted and the corresponding case-notes of these patients were located. The case-notes and the Integrated Clinical Environment (ICE) computer system were hand-searched to allow comparisons of the documented clinical and biochemical information with the information on the request form given to the radiologist.

In order to assess the clinical impact of the after-hours CT A/P scans, the case-notes were further searched to look for documentation of the scan report and documentation of a resulting new management plan. The time of such documentation was noted and compared to the time that the report was listed as being available on PACS. In order to provide tangible data, clinical impact was classified as after-hours CT A/P having the following impact on management: (i) no impact; (ii) ruled out nonacute serious pathology (as determined by clinical question in notes or request form); (iii) ruled out acute serious pathology (as determined by clinical question in notes or request form); (iv) direct impact on medical/surgical management.

Diagnostic utility was largely assessed using clinical outcomes as the gold standard. A true positive (TP) result was considered to be those patients with significant findings on CT A/P, which impacted on management and the patients' symptoms resolved. The false positives (FPs) were those with positive scans that were not borne out by the clinical picture. The true negatives (TNs) were the negative scans where the patients spontaneously improved and the false negatives (FNs) were the negative scans where the patients deteriorated and possibly required intervention. Where the patients proceeded to surgical intervention, the radiological diagnoses were compared to diagnoses based on operative and histopathological findings.

This retrospective project was considered a service evaluation project and thus local or national ethical approval was not sought; however the project was registered with the local Audit Department. Where appropriate, figures are given to 3 significant figures ± standard deviation (SD) or 95% confidence intervals (95% CI) are included.

## 3. Results

The case-notes of 79 of the 96 patients (82.3%) who underwent after-hours CT A/P in the defined time period were obtained and were subsequently considered in the analysis. The 17 sets of notes that could not be accessed were either missing or being used as the patient was being seen as an inpatient or outpatient. No scans were of insufficient quality to be excluded. The mean age of the sample was 55.3 ± 19.3 years (SD) (range 19.7–91.1), with a male : female split of 54.4 : 45.6%.

On analysis for discrepancies between the clinical indication and information given to the radiologist and that in the case-notes or recorded laboratory values, there was no given indication or question in the notes or CT form in 35.4% of cases, no variation was seen in 54.4%, and the remaining 8.9% demonstrated variation as shown in Table 1. The CT A/P scan findings are summarised in Table 2.

The clinical impact is summarised in Table 3(a). Where appropriate, the mean time to such impacts was 7 hours and 48 minutes (range 0 minutes to 23 hours). In the case of the impact 0 minutes after the scan report, it was documented in the notes that the scan had been reported verbally by the radiologist to the surgeon, before being formalised on PACS. Table 3(b) displays the breakdown of the timings of any impact on management.

All 79 report findings were compared to clinical outcomes. There were 44, 4, 26, and 4 TPs, FPs, TNs, and FNs respectively. Of the 26 report findings compared to operative or histopathological findings, there were 19, 5, and 2 TPs, FPs, and FNs, respectively, and no TNs as surgical intervention was not routinely performed in those with negative scans who recovered well. Thus, specificity and negative predictive value (NPV) could not be calculated when comparisons to operative or histopathological findings were made. The diagnostic accuracy of after-hours CT A/P at our institution is otherwise summarised in Table 4 (with 95% CI).

## 4. Discussion

CT A/P is being used increasingly to investigate acute abdominal pain. To our knowledge, there are no studies assessing the clinical impact and diagnostic utility on management of this imaging modality for abdominal pain in an after-hours setting specifically. There was a significant incidence of pathologies picked up on after-hours CT A/P with only a quarter of scans considered normal. The use of after-hours imaging at our institution appears to be justified, with CT scans either having a direct impact on patient management or ruling out a serious acute pathology being queried by the surgical team in over three-quarters of cases. Furthermore, as documentation of any actions based on the CT A/P findings occurred within 4 hours in nearly half of cases and within 12 hours in nearly three-quarters, it would appear the decision to perform such scans after-hours is necessary to make timely decisions on patient management.

When compared to clinical outcomes, the use of after-hours CT A/P at our institution was extremely suitable for ruling in/out acute serious pathologies. When compared to findings at operation or histopathological results, CT A/P demonstrated excellent sensitivity and a good, but slightly reduced, positive predictive value. This suggests that even compared to macroscopic and microscopic findings, after-hours CT A/P can confidently rule out or predict serious pathologies in the majority of cases.

The sensitivity and specificity for CT in the investigation of abdominal pain of unclear origin range between 86.0 and 100% and 79.0 and 97.0% [16, 19–22], respectively. Systematic reviews assessing CT in the work-up of suspected acute appendicitis have calculated the sensitivity between 91.0% and 94% and specificity between 91% and 99% [3, 4]. The majority of these studies used the gold standard of a combination of clinical and operative or histopathological findings for comparison to CT, as was done in this study. Our sensitivity and specificity of 91.7% (79.1–97.3%) and 86.7% (68.4–95.6%), respectively, are comparable with other studies using CT to investigate abdominal pain of uncertain origin. This would suggest that CT retains its accuracy for investigating acute abdominal pain, when used in an after-hours setting in a DGH.

On assessment for any discrepancies in the information given to the radiologist in the request form compared to records there was no apparent clinical indication/question in either the request form or notes in 35.4% of cases. Variation in the information given to the radiologist occurred in 8.9% of cases. These findings are likely to be less significant than it would first seem, as all after-hours CT A/P scans in this study were discussed in person with the on-call consultant radiologist by a clinician at the level of at least a registrar with the request form being provided to the radiographer. The radiologist would likely have been told the key clinical information already. However, in the after-hours setting it remains good practice to clearly document why a scan is being done. One study of 50 patients showed that CT reports are altered 38% of the time, when scans are reinterpreted with clinical information. Furthermore, this study concluded that if inaccurate information is given, it has a detrimental effect on the accuracy of the report [23]. A systematic review looking at multiple diagnostic tests, including abdominal X-rays and CT head scans, concluded that interpreting tests with the clinical information improves the accuracy of such tests [24]. The need to provide accurate clinical information to the radiologist is therefore paramount, particularly as it is likely that, with shift-based work, on-call teams assessing the patients further down the line will be unfamiliar with the patients and will need to be clearly aware of what pathology was being considered.

Limitations of this study include the fact that the sample considered is self-selected. Every effort was made to locate the 17 case-notes that were not available and we would argue that any selection bias is likely to be negligible given that over 80% of the scans in the defined time period were considered. As with most studies of the diagnostic accuracy of abdominal CT, not all patients underwent laparotomy for operative or histopathological diagnosis. This introduced the possibility of work-up bias. However, it would of course be ethically unjustifiable to operate on the patients considered TNs and the gold standard of clinical outcomes provides safe, pragmatic data.

Another possible limitation of our study is that there are no trainee radiologists at our institution with consultant radiologists running the service. In other centres, particularly overnight, radiology registrars may be responsible for interpreting the scans and for seeking consultant opinion when they feel necessary. Reassuringly, a recent UK study, assessing the diagnostic accuracy of CT in patients who underwent emergency laparotomy having presented with an acute abdomen, found no difference in the diagnostic accuracy of the initial registrar CT report compared to consultant reinterpretation [25]. Similarly a study of after-hours reporting by senior registrars demonstrated a comparable 92% accuracy of registrar CT reporting with a higher discrepancy rate actually being made within working hours [26]. We would therefore argue that the results of this study are therefore likely to be largely generalisable to hospitals employing radiology trainees, but of course after-hours imaging should be reviewed in a radiology trainee-based setting to confirm this extrapolation.

It is also important to note that the requests for CT A/P imaging in this study were made by surgical clinicians only who are likely to be able to decide which patients require further imaging more selectively based on their clinical experience. Similar to most hospitals the surgical team usually review patients with an acute abdomen prior to the need for further imaging. As this is a retrospective study we also do not have access to which scans were discussed with the radiologists but deemed not suitable for further imaging. As different radiologists will have different thresholds as to whether to accept a scan request an ideal study would involve strictly defined criteria for accepting scans in the first instance. However such criteria for accepting CT A/P scans do not exist in most hospitals as the complexity of patients often requires the expertise of the radiology and surgical teams to make decisions regarding imaging investigations.

The diagnostic accuracy of CT A/P in the setting of the acute abdomen is well established. The accuracy of the clinical diagnosis of acute abdominal pain is poor [27]. This study demonstrates after-hours accuracy of CT A/P is comparable to within-hours services and has an important effect on clinical management of surgical patients. The more prevalent use of CT A/P to investigate acute abdominal pain may have true benefits for the patient and the National Health Service such as a reduction in hospital stay, negative findings at operation, cost, and even mortality. Further work to compare within and after-hours CT accuracy in the same hospital and time period would be useful. To date, the two randomised controlled trials investigating the early use of CT versus standard diagnostic pathways for acute abdominal pain have been promising but underpowered to detect a reduction in mortality [17, 28]. Further work with adequately powered studies are indicated with long-term follow-up data, to determine the mortality benefit of CT scanning during normal hours and also after-hours services.

## 5. Conclusion

This study demonstrates a high diagnostic utility of after-hours CT A/P scans with reports having a significant impact on clinical management of surgical patients with acute abdominal pain. After-hours CT A/P is therefore justified and necessary service to provide good clinical care for patients. Improvements in providing information when requesting scans are however needed to facilitate accurate reporting.

## Figures and Tables

**Table 1:** Details of the variation in clinical indication or information on CT A/P request form compared to documentation in notes or laboratory records.

Form of variation	Frequency
Differential diagnosis in notes and not request form	3
Differential diagnosis in request form and not in notes	2
Clinical signs/biochemical features in request form not found in notes/laboratory records	2

Total	7 (8.9% of patients)

**Table 2:** An individual breakdown of the after-hours CT A/P findings.

Scan category	Scan finding	Frequency (*n* = 79)	%
Normal	Normal	22	27.8

Acute inflammatory pathology	Appendicitis	12	15.2
	Pancreatitis (uncomplicated)	4	5.1
	Diverticulitis	2	2.5
	Pancreatic necrosis	2	2.5
	Abdominal wall collections	1	1.3
	Descending colitis	1	1.3
	Endometritis	1	1.3
	Ileitis	1	1.3
	Pelvic collection	1	1.3
	Perianal abscess	1	1.3
	Pyelonephritis	1	1.3
	*Total*	*27*	*34.4*

Miscellaneous	Abdominal wall haematoma	1	1.3
	Abdominal wall seroma	1	1.3
	Bilateral inguinal hernia recurrence	1	1.3
	Colonic tumour with metastases	1	1.3
	Ileus	1	1.3
	Ischaemic colitis/tumour	1	1.3
	Marked faecal loading	1	1.3
	Obstructing renal calculus with hydronephrosis	1	1.3
	Oesophageal and abdominal wall varices	1	1.3
	Parastomal and umbilical hernia	1	1.3
	Prostate tumour	1	1.3
	Rectal tumour with metastases	1	1.3
	Subcapsular liver metastases	1	1.3
	Ureteric colic	1	1.3
	*Total*	*14*	*18.2*

Obstruction	Small bowel obstruction: unclear cause	3	3.8
	Small bowel obstruction: abdominal wall hernia	2	2.5
	Small bowel obstruction: small bowel volvulus	2	2.5
	Small bowel obstruction: parastomal hernia	1	1.3
	Large bowel obstruction: abdominal wall hernia	1	1.3
	*Total*	*9*	*11.4*

Perforation	Gastrointestinal tract perforation: unclear site	3	3.8
	Acute perforated appendicitis	1	1.3
	Perforated gallbladder	1	1.3
	Sigmoid perforation	1	1.3
	*Total*	*6*	*7.7*

Inconclusive	Inconclusive	1	1.3

**(a):** 

Description of impact	Frequency (*n* = 79)	%
No impact on management	16	20.3
Ruled out nonacute serious pathology	2	2.5
Ruled out acute serious pathology	22	27.8
Impacted on medical/surgical management	39	49.4

**(b):** 

Time (hours)	Frequency (*n* = 63)	%
<4	30	47.6
4 ≤ *t* < 12	17	27.0
12 ≤ *t* < 24	16	25.4

**Table 4:** The diagnostic accuracy of after-hours CT A/P at our institution.

	Versus clinical outcomes (%)	Versus operative/histopathology findings (%)
Sensitivity	91.7 (79.1–97.3)	90.5 (68.2–90.3)
Specificity	86.7 (68.4–95.6)	—
Positive predictive value	91.7 (79.1–97.3)	79.2 (57.3–92.1)
Negative predictive value	86.7 (79.1–97.3)	—
